# Knowledge about dental care in patients with head and neck cancer among senior dental school students: a cross-sectional descriptive study

**DOI:** 10.1186/s12909-024-05728-0

**Published:** 2024-07-19

**Authors:** Marzieh Karimi Afshar, Milad Behniafar, Elham Abbaszadeh, Molook Torabi Parizi, Mehrnaz Karimi Afshar

**Affiliations:** 1 Orthodontics, Medical Education, Orthodontics Department, School of Dentistry, Kerman University of Medical Sciences, Kerman, Iran; 2 Kerman University of Medical Sciences, Private Practice, Kerman, Iran; 3 Oral Medicine, Oral Disease Department, School of Dentistry, Kerman University of Medical Sciences, Kerman, Iran; 4 Oral and Maxillofacial Pathology, Medical Education, Oral and Maxillofacial Department, School of Dentistry, Kerman University of Medical Sciences, Kerman, Iran; 5 Prosthodontics, Medical Education, Prosthodontics Department, School of Dentistry, Shiraz University of Medical Sciences, Shiraz, Iran

**Keywords:** Dental students, Dental care, Oncology, Chemotherapy, Radiotherapy, Squamous cell carcinoma, Head and neck cancer

## Abstract

**Background:**

The dentist's central role in treating head and neck cancer patients is to care for the patient’s oral cavity before, during, and after radio/chemotherapy. This research aimed to determine dental students' knowledge about head and neck cancer patients’ dental care.

**Methods:**

One hundred and four fifth and sixth-year dental students participated in this cross-sectional descriptive study. The data collection tool was a questionnaire that collected demographic information (gender, year of study) and four questions consisting of attendance to courses or workshops, passing a dedicated course at the university, willing to participate in a course or workshop on the treatment of head and neck cancer patients and self-evaluating information about the treatment of head and neck cancer patients. Final part 36 questions about oral and dental care for head and neck cancer patients before, after, and during treatment. The data were analyzed with SPSS 26 statistical software and using t-tests, ANOVA, and linear regression at the 0.05 significance level.

**Results:**

Most of the participants were in their sixth year (48.9%). A total of 48.1% of the people reported that their information about dental treatments in patients with head and neck cancer was bad. 85% of participants agreed with the necessity of evaluating the patient's mouth and teeth before starting the treatment. The lowest percentage of correct answers was related to the ideal duration of tooth extraction and a poor prognosis before the start of cancer treatment. The regression analysis revealed a significant relationship between years of education, willingness to participate in courses, and students’ knowledge evaluation and knowledge.

**Conclusion:**

The findings showed that students’ awareness of oral and dental treatment and care for patients with head and neck cancer is insufficient. It is recommended that teaching staff pay more attention to the lack of knowledge and effort to educate students by holding special courses and workshops.

## Précis

The dentist's central role in treating head and neck cancer patients is to care for the patient’s oral cavity before, during, and after radio/chemotherapy. This cross-sectional descriptive study aimed to determine dental students' knowledge about head and neck cancer patients’ dental care. The findings of the present study showed that students’ awareness of oral and dental treatment and care for patients with head and neck cancer is insufficient. It is recommended that teaching staff pay more attention to the lack of knowledge and effort to educate students by holding special courses and workshops.

## Introduction

The central role of dentists in treating head and neck cancer patients is to care for the patient’s oral cavity before, during, and after radio/chemotherapy. Due to the interest and in continuation of previous studies about oral and dental health [1–3], this research aimed to determine dental students' knowledge about head and neck cancer patients’ dental care.

Head and neck cancer (HNC) refers to a group of cancers that occur in the oral cavity, pharynx, larynx, paranasal sinuses, nasal cavity, salivary glands, and lymph nodes of the head and neck. HNC is the ninth most common cancer in the world [4]. Oral cancer is one of the major health problems in the world, with a mortality rate of 177,757 out of 377,713 new cases in 2020 and a survival rate of 50% in 5 years [5–7]. HNC treatment includes surgery, radiotherapy, and chemotherapy alone or in combination [8]. HNC and treatment complications often cause important physical problems such as loss of sense of taste, functional problems such as respiratory, speech, and hearing problems, and psychological problems such as depression, social isolation, and delay in returning to work, which hurt all aspects of the affected patient’s life [9, 10]. Oral and dental complications in HNC treatments include mucositis, infection, pain, salivary gland dysfunction, taste change, dysphagia, trismus, and soft and hard tissue necrosis [11–14]. Approximately 80–100% of patients who undergo HNC treatment suffer from oral mucositis. According to the criteria of the World Health Organization, oral mucositis can be grade 3 (severe) or grade 4 (life-threatening) in a large group of patients who receive high-dose radiotherapy. Moreover, it is observed especially in patients with combined radiotherapy and chemotherapy [13]. Delayed side effects of HNC treatment are often irreversible. They may occur several months to several years after the completion of radiotherapy and include trismus, dysphagia, osteoradionecrosis, decreased salivation, permanent dry mouth, and dental caries [14–18]. Dental evaluation and treatment management of HNC patients before and after cancer is one of the cornerstones of a comprehensive care approach [19–21]. The dentist's main role in treating head and neck cancer patients is to take care of the patient's oral cavity before, during, and after radio/chemotherapy. Since oncology patients have a treatment plan that includes different doses of radio or chemotherapy, a careful dental evaluation is needed and the ideal time is before the start of oncological treatment [22]. Poor oral hygiene, and poor dental and periodontal conditions, increase the side effects of HNC treatment, such as non-healing of the wound, and the development of osteoradionecrosis [23]. Dental care before oncological treatment includes oral hygiene instructions, scaling, and root planning, advice to use a non -carious diet, fluoride prophylaxis, and removal of all sources of irritation and infection in the mouth [24].

Good oral hygiene is vital for patients during radiotherapy and chemotherapy. All elective dental treatments should be postponed until the end of treatment [25]. After oncological treatment, the patient needs multiple check-ups. The dentist can monitor the patient by clinical examination to look for possible regional recurrence and metastasis to the cervical region [26]. Dentists should know about the prevention, diagnosis, and management of cancer treatment side effects to minimize the impact of these side effects on patients' lives [22, 27]. Dentists play an important role not only in recognizing precancerous lesions and head and neck cancer but also in recognizing the complications of their treatment and management. This research aimed to determine dental students' knowledge about head and neck cancer patients’ dental care.

## Materials and method

This research was a descriptive cross-sectional study of 104, 5th and 6th-year students of the Faculty of Dentistry of Kerman University of Medical Sciences who were selected through the census sampling method.

The data collected through the questionnaire consisted of 2 parts. The first part was demographic characteristics, (gender and the year of study), and questions: have you attended a course or workshop on the treatment of patients with HNC? Would you like to participate in a course or workshop on the treatment of HNC patients? Have you had a special course on the dental treatment of patients with HNC during your studies in college? How do you evaluate your information about the side effects of chemotherapy and radiation therapy for patients with HNC?

How do you evaluate your information about the side effects of chemotherapy and radiation therapy for patients with HNC? The answers were very good, good, moderate, bad, or very bad.

Part 2 consists of 36 questions about dental care in HNC patients consisting of multiple choice questions in 6 parts a) knowledge of dental treatments,b) knowledge of oral hygiene administration before radiotherapy, c)knowledge about oral side effects of radiotherapy, d)knowledge of causes of dental caries after radiotherapy, e)knowledge about recommendations for patients with xerostomia, f)knowledge about pain control in HNC patients.

After obtaining the necessary permits, a final-year student who had been trained would attend the classroom, and after the explanation of the questionnaire and the purpose of the research, the questionnaire was distributed among the students. If desired, the correct answers were provided to the student after the questionnaire was collected. The approximate time to complete the questionnaire was 10 min.

The way of scoring was that the correct question was given a score of 1 and the wrong answer was given a 0. Therefore, the range of scores was between 0–36. The percentage of correct and incorrect answers was determined for each question, each part of knowledge, and the total knowledge questions. The mean and standard deviation of the knowledge questions were also determined. The percentage of correct answers between 75–100 is good knowledge, 50–74 is average knowledge, and less is considered insufficient knowledge.

The questionnaire was compiled based on texts and articles. The validity of the whole questionnaire was confirmed with a validity coefficient of 0.91 and a validity coefficient of 0.89. SPSS version 26 statistical software and t-tests and ANOVA were used for data analysis. A significance level of 0.05 was used.

The proposal of this project has been registered with the ethics code IR.KMU.REC.1401.601 in the Medical Ethics Committee of Kerman University. The participants were assured that the information in the questionnaires was confidential and that participation in the project was optional.

## Results

In this research, 39.1% of the participants were male, and 60.9% were female. Regarding the year of university admission, 48.9% were in the sixth year. Sixty-three and nine percent of the fifth and sixth-year students had not attended a course or workshop on treating head and neck cancer patients, respectively, and 57.9% were willing to participate in a course or seminar on treating head and neck cancer patients. Forty-eight and one percent of the participants described their information about the side effects of chemotherapy and radiation therapy, respectively, on head and neck cancer patients as bad (Table 1).

**Table 1:** Frequency distribution of participants based on demographic characteristics

Variables	Percent
Gender	
Male	39.1
Female	60.9
Year of entering the university	
Fifth	13.5
Sixth	48.9
Above	37.6
Attendance to courses or workshops on the treatment of head and neck cancer patients	
Yes	36.1
No	63.9
Passing a dedicated course on the treatment of head and neck cancer patients at the university	
Yes	57.9
No	43.1
Willing to participate in a course or workshop on the treatment of head and neck cancer patients	
Yes	80.7
No	19.3
Self-evaluating information about the treatment of head and neck cancer patients	
Very bad	3.0
Bad	48.1
Moderate	42.9
Good	5.3
Very good	0.8

Table 2 shows the knowledge of the participants about dental treatments for patients with head and neck cancers. The most common answer (86.5%) was about the necessity of oral or dental patient evaluation before cancer treatment. The percentage of correct answers to the questions in this section was 49.83%.

**Table 2:** Frequency distributions of HNC patient answers according to dental treatment

Question	% of the correct answers	% of false answers
The necessity of oral /dental patient evaluation before cancer treatments	86.5	13.5
The necessity of oral prophylaxis before cancer treatments	75.9	24.1
An ideal time for subtle oral examination for patients with head and neck cancer	72.9	27.1
The ideal time for radiotherapy after tooth extraction	30.1	69.9
How long do head and neck cancer patients need follow-up after radiotherapy?	40.6	59.4
Which dose of radiotherapy causes osteoradionecrosis?	41.4	58.6
When to stop daily oral and dental hygiene for patients with cancer treatment?	45.1	54.9
If aggressive treatment is necessary for a patient undergoing chemotherapy, what should be the number of platelets before treatment?	55.6	44.4
What is the number of granulocytes in patients under chemotherapy for antibiotic prophylaxis before dental treatment?	53.4	46.6
Symptomatic vital tooth endodontics treatment should be done how many days before the start of cancer treatment?	42.9	57.1
What is the best time to extract teeth with a poor prognosis before cancer treatment?	3.8	96.3

Table 3 shows the oral hygiene instructions provided to patients with HNC before RT. The most common answer (59.4%) was related to the administration of artificial saliva. The percentage of correct answers in this part was 47.44%.

**Table 3:** Percentage of respondents concerning knowledge of oral hygiene administration before radiotherapy

Which of the following administrate before radiotherapy?	% of the correct answers	% of the incorrect answers
Hard toothbrush	42.9	57.1
Artificial saliva	59.4	40.6
Flouride toothpaste	50.4	49.6
Alcoholic mouth wash	40.6	59.4
Non-alcoholic antiseptics	43.6	56.4

Table 4 shows the participants’ knowledge of the oral side effects of radiotherapy, causes of faster tooth decay after radiotherapy and recommendation for patients with xerostomia. The most correct answer to the oral side effects of radiotherapy was a reduction in saliva after radiotherapy. The percentage of total correct answers in this part was 40.5%.

**Table 4:** Percentage of respondents’ knowledge according to oral side effects of radiotherapy, causes of faster tooth decay after radiotherapy and recommendation for patients with xerostomia

Domain	Questions	% of the correct answers	% of incorrect answers
Which of the following are oral /dental radiotherapy side effects?	Many patients experience hypersalivation after radiotherapy	58.6	41.3
	Oral mucosa will be thicker after radiotherapy	41.4	58.6
	The oral mucosa is easily damaged and susceptible to infection	51.9	48.2
	All foods cause damage to the mucosa	21.8	78.2
	Mucositis can increase the risk of oral pain and systemic infection	45.1	54.9
	Oral side effects may lead to radiation dose reduction and treatment discontinuation	27.8	72.2
	Patients undergoing radiation therapy may experience changes in the sense of taste	49.6	50.4
	Radiation dose does not affect the growth and development of children's bones and teeth	24.1	65.9
Which of the following are the causes of faster tooth decay after radiotherapy?	Change in the amount of saliva secretion	70.7	29.6
	Change in the composition of saliva	56.4	43.7
	Using topical fluoride	40.6	59.4
	Change in taste	33.8	66.2
Which of the following is recommended for patients with xerostomia?	Chewing sugar-free chewing gum	57.1	42.9
	Using hot foods for Stimulation of saliva secretion	48.9	51.1
	Using watery to soft foods	52.6	47.4
	Drink water in sips	49.6	50.4

In response to the causes of faster tooth decay after radiotherapy, 70.7% of the participants answered correctly about the change in the amount of saliva secreted, and 56.4% answered about the change in the composition of saliva (Table 4). The percentage of total correct answers in this part was 50.37%.

The participants’ knowledge of the recommendations for patients with xerostomia after HNC treatment is shown in Table 4. The percentage of correct answers in this part was 52.05%.

Regarding pain control, 57.1% of the patients answered correctly about the use of local anesthetics, and 39.8% answered about the use of ice chips (Table 5). The percentage of correct answers in this part was 51.66%.

**Table 5:** Frequency distribution of participants’ knowledge about pain control

Which of the following is recommended for pain control?	% of the correct answer	% of the incorrect answer
Using alcoholic mouthwashes	43.6	58.4
Using topical anesthesia	57.1	42.9
Using ice chips	39.8	60.2
Lubricant the lips	39.1	60.9

The mean and standard deviation of total knowledge was17.59 ± 6.43. There were significant differences according to sex (*P* = 0.05), year of entry to university (*P* = 0.001), tendency to participate in a course or workshop on the treatment of head and neck cancer patients (*P* = 0.011), and knowledge (Table 6).

**Table 6:** Correlations between demographic variables and knowledge

Variable	Mean	Standard deviation	*P* value
Gender			
Male	19.13	6.90	0.05
Female	16.23	5.74	
Year of entering the university			
Fifth year	18.25	6.81	0.001
Sixth year	20.71	6.48	
Above	14.10	4.30	
Attendance to courses or workshops on the treatment of head and neck cancer patients			
yes	18.12	4.87	0.997
no	18.12	7.00	
Passing a dedicated course on the treatment of head and neck cancer patients at the university			
yes	18.92	5.34	0.420
no	17.33	6.61	
Willing to participate in a course or workshop on the treatment of head and neck cancer patients			
yes	20.08	5.84	0.011
no	16.47	6.43	
Evaluating information about the treatment of head and neck cancer patients			
Very bad	15.50	5.91	0.113
bad	16.07	4.53	
Moderate	18.78	7.21	
Good	21.00	9.69	

## Discussion

One of the important approaches to treating patients with HNC is to reduce or eliminate the risk of complications caused by treatment. Dentists should be aware of the importance of preventing, diagnosing, and managing oral complications during treatment to minimize the impact of complications on patients’ lives [22, 27].

In the present study, the range of correct answers was between 40.03 and 52.05 in the different parts. It seems that the knowledge in this study is insufficient, which is compatible with the findings of Pedic et al. [28]. Male students had a much greater level of knowledge in our study. In contrast, other studies have shown that there is no substantial difference in knowledge between men and women [20, 29].

In the present research, sixth-year students had significantly more knowledge. This could be attributed to taking more courses and having more experience working with patients.

In this study, 57.9% wanted to participate in courses or workshops related to the oral/dental care of patients with HNC. A statistically significant difference was observed between the willingness to participate and the awareness score. According to the study of Patel et al. [22], 67% of radiotherapists and 72% of dentists were willing to participate in continuing education courses for oral/dental care in patients with HNC. Given the increase in the number of survivors of HNC due to progress in treatment and supportive care, it is necessary to raise awareness of oral hygiene prevention and treatment to maintain oral health.

In this study alone, 86.5% of the population considered it necessary for patients to have their teeth evaluated before radiotherapy.

According to the study by Pedic et al. [28], 96.7% of Sarajevo students, and in the study by Alqahtani et al. [20] in Saudi Arabia, 97% of people working in the dental profession agreed with the need to evaluate the oral or teeth of patients before radiotherapy, which is almost consistent with the findings of the current study. Oral evaluation before cancer treatment is necessary to prevent and treat dental problems to avoid possible complications during cancer treatment.

In this study, 41.4% of the patients gave correct answers about the radiation dose that led to osteoradionecrosis. The findings of the study by Pedic et al. [28] showed that the percentage of correct answers at 5 universities was between 35.5 and 62.5%, which is similar to the findings of the present study.

Moon et al. showed that the frequency of mandibular osteoradionecrosis is currently a low and modifiable risk factor; for example, tooth extraction before radiotherapy, smoking, and radiotherapy are related to mandibular osteoradionecrosis [15].

In the present study, the students answered questions about their platelet and leukocyte counts if they needed treatment during cancer treatment (55.6% and 53.4%, respectively). According to Pedic et al.'s study [28], 80% of Sarajevo students and 76.4% of Zagreb students answered correctly, which is more than what was observed in the present study.

Patients who are receiving chemotherapy without radiotherapy can undergo dental treatment if their blood count is stable (leukocytes at least 2000 cells/mm^3^, neutrophils more than 1000 cells/mm^3^, and platelets more than 50,000 cells/mm^3^. On the day of dental treatment, a complete blood test and differential blood count (DBC) are required [25].

In the present study, only 3.8% of the participants were aware of the best time to remove teeth with a poor prognosis before HNC treatment, which is less than the prevalence reported by Pedic et al. [28] and Alpöz et al. [21] studies that 65.7% and 27.3%, respectively, of the students, knew that teeth with a poor prognosis should be extracted at least 3 weeks before starting the treatment respectively.

In the present study, 42.9% of people answered correctly about the duration of endodontic treatment for symptomatic vital teeth before cancer treatment. In Pedic et al.'s study [28], 59.3% of the participants answered correctly.

The best time for dental treatment was at least three weeks before the start of oncological treatment. If the patient does not have an acute infection, tooth extraction should be performed after radiotherapy and during the “golden window period” [23].

The knowledge of students about the materials and devices prescribed for maintaining oral and dental hygiene was insufficient. Only 42.9% of people were aware of proper toothbrush prescriptions, and 43.6% were aware of alcohol-free antiseptics. According to the study by Alqahtani et al. [20], 59.5% were aware of proper toothbrush use, and 94% of alcohol-free antiseptics were prescribed; these findings are more than those of the present study. The guidelines established by the Multinational Association of Supportive Care in Cancer and the International Society of Oral Oncology recommend the use of a soft toothbrush, waxed dental floss, and several mouthwashes after brushing [24].

One of the main guidelines in the management of patients with HNC who are receiving radiotherapy or are going to undergo radiotherapy is the use of mouthwash or topical products without alcohol [25–27].

In this study, 27.8% of people answered correctly that oral complications may lead to dose reduction or temporary discontinuation of radiotherapy. In the study by Alqahtani et al. study [20], 29% of the participants did not know about this matter, which is consistent with our study.

When severe oral complications are observed during radiotherapy, it is necessary to temporarily stop radiotherapy [20, 29].

In this study, 51.8% of participants were aware of not prescribing spicy foods to patients with dry mouths. In the study of Alqahtani et al. [20] in Saudi Arabia, 94% of people were aware of this. The reason for this difference in the population is studied because, in the study of Samim et al. [26], dentists and dental specialists were studied.

In this research, 24.1% of people answered correctly that radiation dose affects the growth and development of children's bones and teeth. Many dental and developmental complications of maxillary bones during radiotherapy depend on age, radiation dose, and radiation location. The information of the students is insufficient in this matter.

In this study, only 45.1% of people gave the correct answer to the question of when to stop daily oral hygiene in patients undergoing cervical cancer treatment. Considering the changes in the quantity and quality of saliva and the change in the sense of taste of people receiving radiotherapy, which leads to dry mouth and an increase in the incidence of dental caries, daily oral and dental hygiene should never be stopped in patients with HNC.

Forty-six percent of the people in this study gave the correct answer to the time required for the follow-up of HNC patients after radiotherapy, which is less than that in the study by Alqahtani et al. [20] where 80% of the participants gave the correct answer. Follow-ups after radiotherapy should be performed at least once every 3–4 months to control caries, control saliva flow, and control periodontal diseases, as well as health education and encouragement of the patient not to use cariogenic foods.

Limitations: Since the questionnaires were completed by the participants, there was a possibility that some answers were not accurate, which was beyond the researcher's control.

## Conclusion

The findings of this study showed that students' awareness of oral/dental care in patients with HNC was insufficient. There were statistically significant relationships between knowledge, years of education, sex, and willingness to participate in special oral care courses for patients with HNC. The student’s knowledge about the best time for tooth extraction before radiotherapy was very low. Due to insufficient students’ knowledge, it is recommended that a workshop or course on dental treatments be held for HNC patients.

## Data Availability

Data is provided within the manuscript.
